# Comparison Between Adalimumab and Infliximab in Perianal Crohn’s Disease: A Systematic Review and Meta-Analysis

**DOI:** 10.1016/j.gastha.2025.100697

**Published:** 2025-05-09

**Authors:** Tarek Aboursheid, Azizullah Beran, Mohamad Hijazi, Mhd Kutaiba Albuni, Bisher Sawaf, John J. Guardiola, Khaled Abdeljawad, Benjamin D. McDonald, David T. Rubin

**Affiliations:** 1 Department of Internal Medicine, Saint Francis Hospital, Evanston, Illinois; 2 Division of Gastroenterology and Hepatology, Indiana University, Indianapolis, Indiana; 3 Department of Internal Medicine, Trihealth Good Samaritan Hospital, Cincinnati, Ohio; 4 Department of Internal Medicine, The University of Toledo, Toledo, Ohio; 5 Inflammatory Bowel Disease Center, University of Chicago Medicine, Chicago, Illinois

**Keywords:** Adalimumab, Infliximab, Perianal Crohn’s disease, Anti-TNF, Fistula

## Abstract

**Background and Aims:**

Anti–tumor necrosis factor therapy has been a mainstay of medical management of perianal Crohn’s disease (CD). This systematic review and meta-analysis aimed to compare the efficacy of adalimumab (ADA) and infliximab (IFX) for the treatment of perianal fistulizing CD.

**Methods:**

We searched PubMed, Embase, and Web of Science databases for studies that compared the efficacy of ADA with IFX for treatment of patients with perianal fistulizing CD. Single-arm studies were excluded. The primary outcomes were perianal CD healing evaluated clinically or radiologically.

**Results:**

Six observational studies with a total of 590 patients with perianal CD (216 received ADA and 374 received IFX) were included. Overall, perianal CD healing was comparable between ADA and IFX (risk ratio (RR) 0.90, 95% confidence interval (CI) 0.76–1.06, *P* = .20). Subgroup analysis based on the healing assessment method showed no significant difference between ADA and IFX whether assessment was performed clinically (RR 0.90, 95% CI 0.67–1.21, *P* = .49) or radiologically (RR 0.91, 95% CI 0.74–1.13, *P* = .40).

**Conclusion:**

In this systematic review and meta-analysis, ADA was not inferior to IFX in effectiveness for the treatment of perianal CD. Head-to-head prospective and randomized controlled studies comparing ADA and IFX are necessary to validate the findings of this study.

## Introduction

Crohn’s disease (CD) is a chronic inflammatory bowel disease that can affect any part of the gastrointestinal tract from the mouth to the anus. It is characterized by transmural inflammation that has the potential to form fistulas connecting the bowels to any adjacent organs, including the skin.^1^ The term Perianal CD refers to the involvement of the perianal area, which can include perianal fistulas, perianal abscesses, anal strictures and anal fissures.^2^ It is estimated that approximately 20% of CD patients will develop perianal disease (abscess, fistula or both) within the first decade of diagnosis. Additionally, around 11.5% of CD cases present with perianal involvement at the time of initial diagnosis.^3^ Perianal fistulas are the most common type of perianal disease and one of the most difficult complications of CD to treat, often leading to significant morbidity and a decline in quality of life.^4^^,^^5^

Infliximab (IFX) and adalimumab (ADA) are both anti–tumor necrosis factor (TNF) monoclonal antibodies that are frequently used by patients with perianal CD. Despite their widespread use, there are limited comparative efficacy data to guide clinical decision making. Indeed, while no randomized controlled trials (RCTs) have directly compared these 2 medications against each other, existing evidence offers some insights. A previously published network meta-analysis indirectly comparing IFX and ADA found comparable efficacy in achieving remission in fistulizing CD.^6^ However, there is a need for effectiveness data from direct comparative studies. We conducted a systematic review and meta-analysis of studies that directly compared ADA and IFX in managing perianal CD.

## Methods

This systematic review and meta-analysis was performed based on the guidelines of the Preferred Reporting Items for Systematic reviews and Meta-analysis^7^ and the guidelines of Meta-analysis of Observational Studies in Epidemiology.^8^

### Search Strategy

We conducted a comprehensive literature search of PubMed, Embase, and Web of Science databases from inception through August 31, 2024. The search terms used were (“infliximab” or “adalimumab”) and (“fistulizing Crohn's disease" or "perianal Crohn's disease”). We also manually searched for more studies referenced in the relevant articles. Table A1 shows the full search terms used in each database.

### Inclusion and Exclusion Criteria

We included peer-reviewed studies that compared ADA with IFX in patients with biologic-naive perianal fistulizing CD. Single-arm studies and non-English studies were excluded. Two investigators (T.A. and M.H.) independently performed the literature search and shortlisted the studies for final review. Discrepancies were resolved by a third investigator (A.B.).

### Data Extraction

We extracted all the relevant study and patient characteristics and outcome measures from the included studies. Study characteristics included country of origin, design, sample size in each group, and reported outcomes (fistula healing either clinically or radiologically). Extracted patient characteristics included mean age, male sex percentage, duration of treatments, seton placement, and use of steroids and immunomodulators.

### Outcomes and Definitions

The primary outcome was overall perianal CD healing, which was defined as perianal CD healing by clinical or radiological assessment. Subgroup analysis was performed based on perianal CD healing assessment method (clinical vs radiological healing) and the degree of radiological healing (partial vs complete radiological healing).

Since there is currently no consensus on the definition of radiological healing for perianal CD,^9^ we defined complete radiological healing as complete closure of the fistula on magnetic resonance imaging (MRI) or a Van Assche Index of 0. Partial radiological healing was defined as at least a 50% reduction in the number of fistulous tracts, a Van Assche Index score of 6 or less on MRI, or at least a 50% reduction in the Van Assche Index from baseline. Overall, radiological healing encompassed both complete and partial radiological healing. Clinical healing was defined as fistula closure or cessation of drainage as assessed clinically.

### Statistical Analysis

The pooled risk ratio (RR) and the corresponding confidence intervals (CIs) for the desired outcomes were calculated using the random-effects model and Mantel–Haenszel method. A *P* value of <0.05 was considered statistically significant. Statistical heterogeneity was assessed using the I^2^ statistic as defined by the Cochrane handbook for systematic reviews, and an I^2^ value of ≥ 50% was considered significant heterogeneity. All statistical analyses were performed via Review Manager 5.4 software.

### Bias Assessment

The Newcastle Ottawa Quality Assessment Scale was used to assess the quality of the studies based on the selection of the study groups, comparability of study groups, and ascertainment of exposure/outcome.^10^ Studies with total scores of ≥6 were considered to have a low risk of bias. Two reviewers (T.A. and M.K.A.) independently assessed each study for bias. Discrepancies were resolved by a third reviewer (A.B.).

## Results

### Study Selection

**Figure 1:**
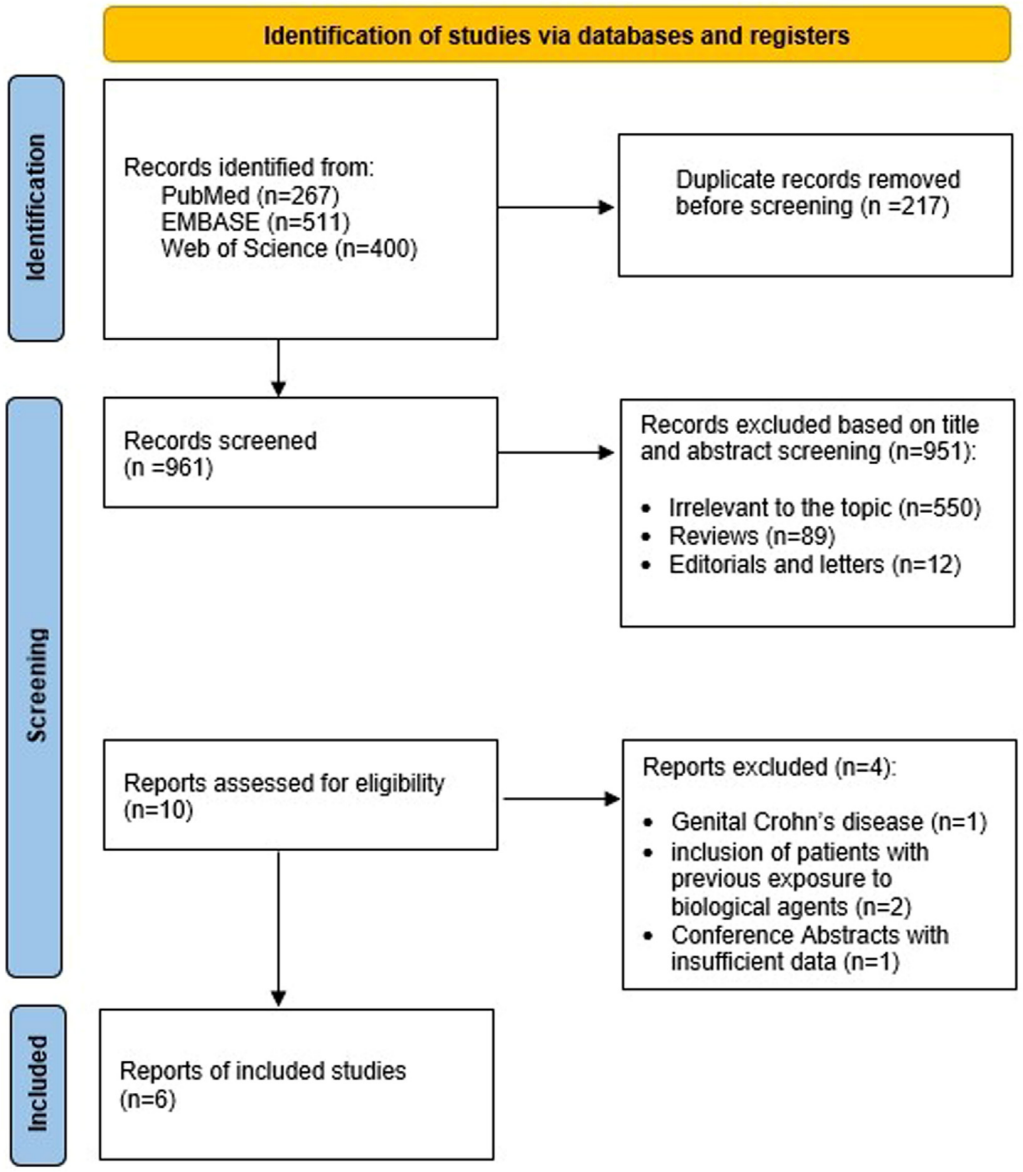
PRISMA flow diagram for the selection of studies. PRISMA, Preferred Reporting Items for Systematic reviews and Meta-Analysis.

We identified and screened 1178 studies; 10 met the eligibility criteria for this systematic review. Among these 10 studies, 6^11^, ^12^, ^13^, ^14^, ^15^, ^16^ met the eligibility criteria and were included for the meta-analysis and 3 were excluded due to inclusion of biological-experienced patients or CD patients with genital fistulas. One conference abstract was excluded due to insufficient data. The detailed selection process is summarized in Figure 1.

### Study and Patients’ Characteristics

Study and patient characteristics are summarized in Table. All studies were published between 2016 and 2023. Five were retrospective cohort studies 11, 12, 13, 14, 15 and 1 was a prospective cohort study.^16^ Regarding country of origin, 3 studies were conducted in Australia,^12^^,^^14^^,^^15^ 1 in Canada,^16^ 1 in Saudi Arabia,^11^ and 1 in the USA.^13^ A total of 590 patients with perianal CD (216 received ADA and 374 received IFX) were included. All the patients had received an anti-TNF agent for at least 1 year at the time of evaluation. The doses of ADA and IFX administered in each study, along with the dose escalation method for initial nonresponders, are detailed in Table A2. The combination therapies reported by the studies included azathioprine, 6-mercaptopurine, methotrexate, seton placement, and/or steroids. However, information on the rate of use of these treatments alongside IFX or ADA was limited. Table shows the combination therapies reported in each study. Four studies^13^, ^14^, ^15^, ^16^ evaluated perianal Fistulizing CD healing clinically while 2 studies^11^^,^^12^ assessed the healing radiologically using (MRI).

**Table:** Studies and Patients Characteristics

Studies and patients characteristics	Azzam 2019	Gregorio 2021	Gu 2022	Maas 2023	Narula 2016	Varma 2016
Study design	RC	RC	RC	RC	PC	RC
Country of origin	KSA	Australia	Australia	USA	Canada	Australia
Number of patients, total	61	193	114	151	34	37
IFX, n (%)	42 (69)	117 (61)	66 (58)	92 (61)	32 (94)	25 (68)
ADA, n (%)	19 (31)	76 (39)	48 (42)	59 (39)	2 (6)	12 (32)
Males, n (%)	36 (59)	112 (58)	72 (63.1)	90 (59.6)	-	-
Age, mean (SD)	33.3 (12.1)	36.4 (12.8)	37.1 (14.0)	29.4 (12.5)	-	-
Duration of therapy, mo	12	IFX [30 (9.6–56.4)]^b^ ADA [25.2 (12–44.4)]^b^	IFX [33.6 (18.6–5.3)]^b^ ADA [42 (25–72.2)]^b^	12	12.4, (1.5)^c^	12
Seton placements, total (IFX/ADA)	12	-	-	76 (49/27)	-	-
Immunomodulators^a^, total (IFX/ADA)	51	128 (84/44)	75 (46/29)	37 (23/14)	-	-
Steroids, total (IFX/ADA)	9	1 (0/1)	-	-	-	-

PC, prospective cohort; RC, retrospective cohort.

a Immunomodulators included azathioprine, 6-mercaptopurine and methotrexate.

b Median and (Interquartile range).

c Mean, (standard deviation).

### Primary Outcome

**Figure 2:**
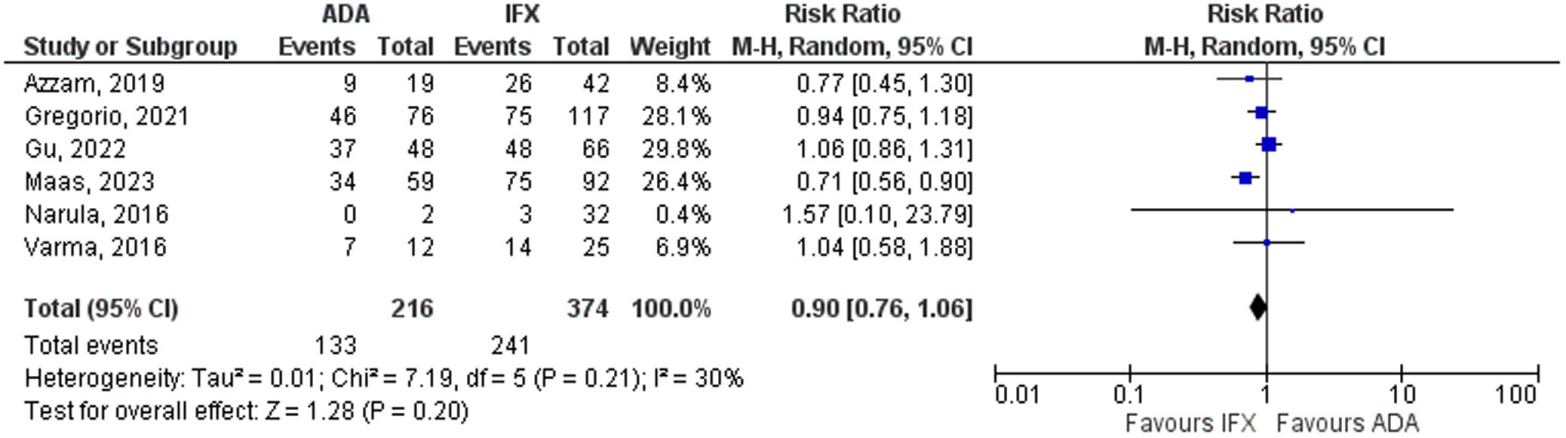
Overall perianal CD healing.

Overall, perianal CD healing was comparable between ADA and IFX groups (61.5% vs 64.4%, respectively) (RR 0.90, 95% CI 0.76–1.06, *P* = .20), with I^2^ = 30% suggesting low heterogeneity between the included studies (Figure 2).

### Subgroup Analyses

**Figure 3:**
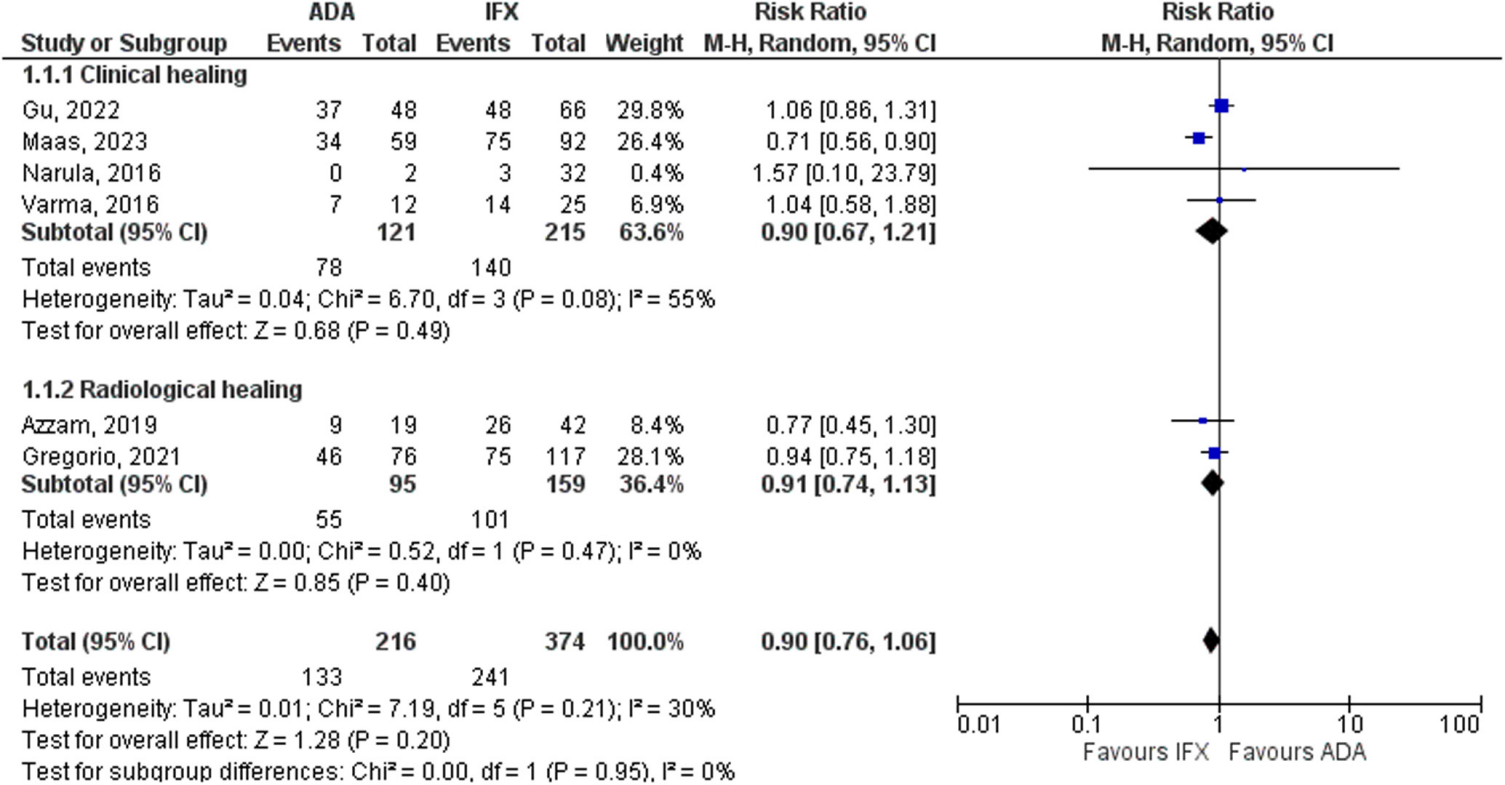
Subgroup analysis based on the method of assessment of perianal CD healing.

Subgroup analysis based on the perianal CD healing assessment method showed that there was no significant difference between ADA and IFX groups whether assessment performed clinically (64.4% vs 65.1%, respectively; RR 0.90, 95% CI 0.67–1.21, *P* = .49, I^2^ = 55%) or radiologically (RR 0.91, 95% CI 0.74–1.13, *P* = .40, I^2^ = 0%) (Figure 3).

**Figure 4:**
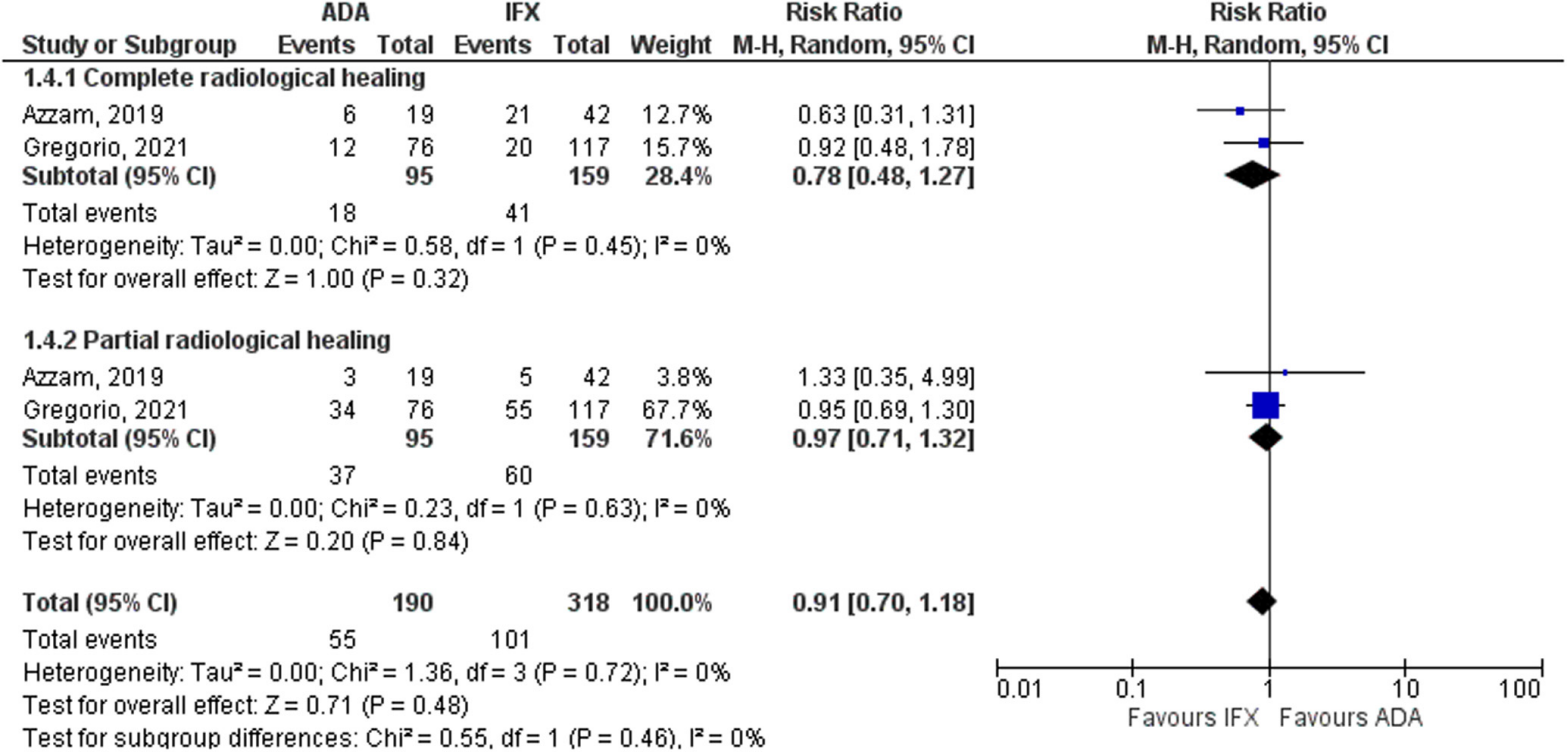
Subgroup analysis based on complete and partial radiological healing of perianal CD.

In terms of radiological assessment, subgroup analysis showed no significant differences between the 2 groups in achieving partial (RR 0.97, 95% CI 0.71–1.32, *P* = .84, I^2^ = 0%) or complete (RR 0.78, 95% CI 0.48–1.27, *P* = .32, I^2^ = 0%) healing of perianal CD (Figure 4).

### Bias Assessment

The risk of bias for each included study is summarized in Table A2. All the 6^11^, ^12^, ^13^, ^14^, ^15^, ^16^ included studies were at low risk of bias (Table A2).

## Discussion

Perianal fistulas represent a substantial burden in CD, constituting approximately 54% of all fistulas associated with the condition. Several risk factors contribute to the development of perianal fistulizing CD, including colonic disease—especially rectal involvement—youthful age at diagnosis, male gender, extended disease duration, and the presence of extraintestinal manifestations.^17^ The pathogenesis of fistulas in CD is complex and not well understood. It is believed to happen in genetically predisposed individuals with impaired immune response to gut flora. This dysregulated immune response triggers the production of various cytokines, including TNF α, interleukin 13, and transforming growth factor-β, contributing to the formation and perpetuation of fistulas.^18^^,^^19^

The treatment of perianal fistulas in CD poses significant challenges and requires a multidisciplinary approach with nearly one-third of patients experiencing recurrence despite therapeutic interventions.^18^ Surgical options include incision and drainage of perianal abscesses, setons placement to prevent the recurrence of new abscesses and fistulas, fistulotomy for low intersphincteric fistulas, and ligation of the intersphincteric fistula tract and endorectal advancement flap. Although antibiotics play a role in managing perianal fistulas and abscesses, they are generally insufficient as monotherapy as most fistulas tend to recur after antibiotic cessation. Immunomodulators, such as Azathioprine and 6-mercaptopurine, have demonstrated limited efficacy when used alone for perianal CD.^17^ Recent research has also explored the use of mesenchymal stem cells in the treatment of perianal fistulas. A phase 3 RCT evaluated darvadstrocel, an allogeneic mesenchymal stem cell therapy, in patients with complex perianal fistulas. However, the study found that darvadstrocel did not significantly outperform placebo at 24 and 52 weeks in terms of fistula closure .^20^ The evidence supporting the effectiveness of Ustekinumab, an interleukin-12/23 antagonist, and Vedolizumab, a gut-selective anti-α_4_β_7_ integrin, in treating perianal CD is limited, primarily derived from subgroup analyses of RCTs. In contrast, anti-TNF a agents appear to have the strongest evidence supporting their first-line use in treating perianal CD.^17^

IFX, an anti-TNF chimeric monoclonal antibody, stands as the first-line treatment for perianal fistulizing CD, being the first and most studied biological medication in perianal CD. Traditionally, IFX is administered via intravenous infusion; however, a subcutaneous formulation has recently been approved, although it is not yet widely adopted in clinical practice. Conversely, ADA, a fully humanized anti-TNF α monoclonal antibody approved primarily for the management of luminal CD and is administered subcutaneously.

Despite sharing a common mechanism of action with IFX, ADA's efficacy specifically in fistulizing perianal CD has not been studied comprehensively. Nonetheless, post hoc analyses derived from various studies have unveiled superior efficacy of ADA compared to placebo in perianal CD. These findings have propelled its integration into clinical practice.^17^^,^^21^

Given the absence of RCTs directly comparing IFX and ADA, prior network meta-analyses indirectly assessed their efficacy in treating fistulizing perianal CD, revealing comparable efficacy in achieving remission between the 2 medications.^6^ Similarly, in this meta-analysis of 6 comparative studies that included 590 patients with fistulizing perianal CD, our results showed that ADA is noninferior to IFX in terms of effectiveness.

Four of the included studies evaluated healing clinically. The study by Gu et al.^14^ aimed to determine the effect of IFX and ADA trough levels on perianal fistula healing. both treatment groups received similar rates of immunomodulators overall. Their findings indicated that maintaining higher trough levels was correlated with improved fistula healing rates. Our analysis of the reported healing rates of IFX and ADA in this study showed no significant difference between the 2 medications.

Varma et al.^15^ conducted a retrospective study comparing IFX with ADA in CD which also showed similar efficacy between ADA and IFX perianal CD. However, the reporting of perianal disease healing as a subcategory limited the availability of detailed information regarding patients' demographics and concurrent treatments. Despite that, the authors reported no statistically significant differences in the rates of fistula healing among patients undergoing anti-TNF monotherapy or combination therapy with either agent. Nonetheless, IFX was associated with a reduction in perianal fistula-related hospitalizations. The authors noted that a potential source of bias in this result is that patients receiving IFX were evaluated every 8 weeks due to IFX intravenous infusion delivery method, which could have facilitated earlier detection of fistula-related complications compared to patients on ADA, who typically administer the medication subcutaneously at home.

Similarly, Narula et al.^16^ conducted a prospective observational study comparing IFX and ADA in anti-TNF naïve patients. In this study, only 2 patients with perianal disease received ADA compared to 32 patients who received IFX. Both of those patients receiving ADA did not achieve clinical healing, but due to the small sample size in the ADA group, meaningful comparisons regarding efficacy between the 2 agents were limited.

The study conducted by Maas et al.^13^ stands out as the sole investigation reporting a higher efficacy associated with IFX over ADA. However, during the 12-month therapy period, a significantly greater proportion of patients in the IFX group underwent dose escalation compared to those in the ADA group. Given the established correlation between higher levels of anti-TNF agents and improved fistula closure,^12^^,^^14^^,^^22^ this greater frequency of dose escalations in the IFX cohort might have contributed to elevated drug levels in the IFX group, potentially driving superior fistula closure rates compared to ADA.

Recognizing the superior accuracy of MRI in assessing fistula healing compared to clinical examination,^23^ we conducted a separate analysis focusing on studies evaluating radiological healing of perianal disease. Perianal CD healing was evaluated by MRI in 2 of the included studies. Azzam et al^11^ retrospectively evaluated the healing of perianal fistulas after 12 months of anti-TNF therapy. In addition to showing similar healing rates between IFX and ADA, the authors also conducted a multivariable analysis on the predictors of response to treatment in patients receiving anti-TNF for perianal CD. They found that low baseline body mass index (BMI) was a predictor of poor fistula closure. Conversely, the study by De Gregorio,^12^ which primarly aimed to evaluate perianal fistula healing using MRI correlated to anti-TNF levels, found that higher BMI was independently associated with poor radiological healing. More studies are needed to evaluate the relationship between BMI and response to anti-TNF therapy in patients with perianal disease.

Our study has important limitations. First, all 6 included studies were observational. Of these, 5 were retrospective cohort studies and 1 was a prospective cohort study. Second, limited detailed data were available on the use of combination therapy as well as the development of anti-TNF antibodies, which could have provided crucial insights into treatment resistance patterns. Third, data on drug levels and complexity of the perianal disease were limited and could have affected the response to treatment in each group. Fourth, we could not conduct a leave-out sensitivity analysis and publications bias analysis due to the small number of included studies (<10). Finally, it is a general limitation across the perianal disease state that validated objective measures of perianal disease activity and healing are not fully developed.^9^

Our study has several strengths despite its limitations. First, this is the first meta-analysis to conduct a direct comparison between ADA and IFX for the treatment of fistulizing perianal CD. Second, we conducted an additional analysis focused on studies that assessed the healing of perianal CD through radiological evaluations and found consistent results. Finally, all studies included in our analysis were determined to have a low risk of bias.

## Conclusion

Our study demonstrated that ADA was not inferior to IFX in effectiveness in treating perianal CD. ADA may be an acceptable alternative to IFX in these patients. Large-scale head-to-head prospective studies and RCTs comparing ADA and IFX for treatment of perianal CD are necessary to validate our findings.
