# Supplementary material for: Comparison Between Adalimumab and Infliximab in Perianal Crohn’s Disease: A Systematic Review and Meta-Analysis
Source: Gastro Hep Adv. 2025 May 9;4(8):100697. doi: 10.1016/j.gastha.2025.100697 (PMC12221630; doi:10.1016/j.gastha.2025.100697)
Supplement: Supplementary Material [file mmc1.docx]

# **Supplementary Appendix**

**Comparison between Adalimumab and Infliximab in Perianal Crohn’s Disease: A Systematic Review and Meta-Analysis**

**Supplementary Tables**

| Title | Page |
| --- | --- |
| Supplementary Table 1: Search strategy used in each database searched. | 2 |
| Supplementary Table 2: Doses and dose-escalation methods in the included studies. | 3 |
| Supplementary Table 3: Quality assessment of the included studies in the meta-analysis. | 4 |

Supplementary Table 1: Search strategy used in each database searched.

| Database | Search Strategy | Articles retrieved |
| --- | --- | --- |
| PubMed | ("infliximab"[MeSH Terms] OR "infliximab"[All Fields] OR "infliximab s"[All Fields] OR ("adalimumab"[MeSH Terms] OR "adalimumab"[All Fields])) AND ("fistulizing crohn's disease"[All Fields] OR "perianal crohn's disease"[All Fields]) | 267 |
| Embase | ('infliximab'/exp OR infliximab OR 'adalimumab'/exp OR adalimumab) AND ('fistulizing crohns' OR 'perianal crohns') | 511 |
| Web of Science | (infliximab OR adalimumab) AND ("fistulizing crohn's disease" OR "perianal crohn's disease") | 400 |

Supplementary Table 2: Doses and dose-escalation methods in the included studies.

| Study |  | Induction | Maintenance | Escalation method |
| --- | --- | --- | --- | --- |
| Azzam 2019 | IFX | 5mg/kg at weeks 0, 2, and 6 | - | decreasing the interval or increasing the dose. |
|  | ADA | 160, 80, and 40 mg at weeks 0, 2, and 4 | - | decreasing the interval or increasing the dose |
| Gregorio 2021 | IFX | - | 5 mg/kg every 8 weeks | decreasing the interval or increasing the dose |
|  | ADA | - | 40 mg every 2 weeks | decreasing the interval or increasing the dose |
| Gu 2022 | IFX | 5 mg/kg at weeks 0, 2, and 6 | - | dose escalation (5, 7.5, 10, 15 or 20 mg/kg every 6 or 8 weeks). |
|  | ADA | 160 mg at week 0, 80 mg at week 2 | - | dose escalation (40 mg weekly) |
| Maas 2023 | IFX | - | - | - |
|  | ADA | - | - | - |
| Narula 2016 | IFX | 5 mg/kg at week 0, 2 and 6 | 5/mg/kg every 8 weeks | decreasing the interval or increasing the dose. |
|  | ADA | 160 mg at week 0, 80 mg at week 2 | 40 mg every 2 weeks | decreasing the interval to weekly |
| Varma 2016 | IFX | - | - | - |
|  | ADA | - | - | - |

IFX: infliximab, ADA: adalimumab

Supplementary Table 3: Quality assessment of the included studies in the meta-analysis.

| Observational studies | Selection | | | | Comparability | Outcome | | | Overall risk of bias |
| --- | --- | --- | --- | --- | --- | --- | --- | --- | --- |
|  | Representativeness of the exposed cohort | Selection of non-exposed cohort | Ascertainment of exposure | Demonstration that outcome of interest was not present at start of study | Comparability of the cohorts on the basis of design or analysis | Assessment of outcome | Was follow up long enough for outcomes to occur | Adequacy of follow up cohorts |  |
| **Azzam 2019** | 1 | 1 | 1 | 1 | 0 | 1 | 1 | 1 | 7 |
| **Gregorio 2021** | 1 | 1 | 1 | 1 | 1 | 1 | 1 | 1 | 8 |
| **Gu 2022** | 1 | 1 | 1 | 1 | 1 | 1 | 1 | 1 | 8 |
| **Maas 2023** | 1 | 1 | 1 | 1 | 1 | 1 | 1 | 1 | 8 |
| **varma2016** | 1 | 1 | 1 | 1 | 0 | 1 | 1 | 1 | 7 |
| **Narula 2016** | 1 | 1 | 1 | 1 | 0 | 1 | 1 | 1 | 7 |
| **Azzam 2019** | 1 | 1 | 1 | 1 | 0 | 1 | 1 | 1 | 7 |
